# Changes in attitudes of mental health care staff surrounding the implementation of peer support work: A qualitative study

**DOI:** 10.1371/journal.pone.0319830

**Published:** 2025-04-01

**Authors:** Guillermo Ruiz-Pérez, Madeleine Küsel, Sebastian von Peter

**Affiliations:** 1 Department of Psychiatry and Psychotherapy, Brandenburg Medical School, Immanuel Klinik Rüdersdorf, Rüdersdorf, Germany; Vellore Institute of Technology, INDIA

## Abstract

**Background:**

The integration of Peer Support Workers (PSWs) in mental health settings may encounter resistance due to the attitudes of Mental Health Workers (MHWs). At the same time, PSWs may initiate changes in the attitudes, as well as in the larger institutional culture. This paper addresses the question of which changes happen and how they occur.

**Methods:**

This work is part of the ImpPeer-Psy5 study, funded by the German Innovation Fund, which examines the nationwide implementation of PSWs in health insurance-funded mental health care. Data were derived from two qualitative sub-studies: QUAL1encompassed 57 problem-centered interviews conducted with PSWs, MHWs working together with PSWs, and service users; QUAL2 consisted of one focus group and five interviews only with MHWs. Qualitative data were analyzed using a modified thematic analysis adapted to a collaborative research approach.

**Results:**

The main findings included that working with PSWs makes MHWs better integrate lived experience insights into their caregiving, pay more attention to life contexts, advance towards a more appreciative language, use diagnostic categories more flexibly, and show more openness towards their own affectedness. These changes are reported to be mediated by the simple presence of PSWs, their “role model” function for other staff, or more direct forms of feedback and critique.

**Conclusion:**

Overall, staff attitudes changed as a result of reflexive processes induced by their interactions with PSWs. This poses a dilemma as changes in MHWs and larger institutions are expected to occur prior to the implementation of peer support work to facilitate their integration. Further research is needed to evaluate whether and how staff training can facilitate the implementation of PSWs by targeting MHWs’ attitudes.

## Introduction

Although there is evidence of the presence of peer support workers in mental health services at the end of the 18th century [1], peer support re-emerged as a group movement primarily driven by users outside of and as a critical alternative to mental health care services [2]. Despite their origin, over the past decades, peer support workers (PSWs) have also been employed in the mental health care sector, leading to controversies on how to implement this originally resistant workforce in the context of more mainstream services [3–5]. These debates were substantiated by research on the effects and benefits of implementing peer support for users [6–8]. Mounting evidence suggests that peer support work leads to increased user engagement and participation [9,10], heightened satisfaction among users [11,12], and reduced hospitalizations or alleviation of symptom severity [13–16]. Furthermore, several authors contend that the implementation of PSWs facilitates the shift towards a more recovery-oriented perspective on mental health crises [17–20] and a more comprehensive approach to mental health care [8,21].

Despite these benefits, various obstacles hinder the successful implementation of peer support work in the context of mental healthcare services [22]. One of the most significant barriers is the attitude of MHWs, especially those who adhere to the medical model of mental health care [22–25], which highlights how MHWs’ tendencies to medicalize diagnoses can obstruct the work of PSWs and stigmatize this workforce [22]. Moreover, MHWs’ attitudes can also impede interactions with PSWs, reinforce hierarchies, and shape opinions about what constitutes proper care [26], ultimately influencing the PSWs’ job satisfaction [5,27,28] efficiency, and overall professional development [5,22,29].

Implementing peer support requires both micro-level changes in individual attitudes and meso-level institutional transformations [30]. A recent systematic review concluded that institutional culture is the most influential factor in the successful implementation of peer support [5]. Correspondingly, Ramesh et al. highlighted the importance of staff attitudes as influential as long as they can intervene as facilitators or barriers to implementation [31]. In addition, understanding the attitudes toward PSWs requires attention to contextual issues such as socioeconomic circumstances, previous interactions with PSWs, and how the integration of peer support is planned within the organizational culture [32].

Without changes on these levels, a ‘collision between worlds’ may occur, resulting in conflicts between MHWs and PSWs due to irreconcilable differences in attitudes or cultural dissonance [23]. In this context, both Davidson [1] and Haun et al. [33] emphasized the need to change the institutional environment prior to the implementation of PSWs. Professional mental health teams trained in recovery values —such as connectedness, empowerment and personal responsibility, hope and identity, as described by the CHIME framework [34]—and focusing on helping individuals live meaningful lives beyond the label of mental illness may facilitate the integration this workforce [25,27,28]. Further recommendations [35] highlight the importance of MHWs’ training and also internships [36–39] to foster attitudes closer to the recovery model of mental health care [21,26,40,41]. Aguey-Zinsou et al. even suggest that such training should be incorporated into undergraduate education [21], whereas other authors propose additional preparatory measures that are crucial for the successful collaboration of PSWs and other staff [42].

At the same time, PSWs are often called “change agents” as they may motivate perspective changes among the staff members, they a collaborating with [36,43,44]. Working with PSWs, first, provides MHWs with more knowledge of users’ experiences, also enhancing their competence in communicating and listening [25]; for instance, turning the MHWs’ language into a less managerial or technical one and reducing micro-aggressive or stigmatizing forms of communication [45,46]. Both may also reflect the power relations that occur between professional staff and patients [47], often resulting into a “them-and-us” division between these groups, a division that may be reduced by PSWs that constitute a case that goes beyond [48,49]. Similarly, Byrne et al. argued that the implementation of PSWs supports a culture of disclosure on the side of MHWs [50].

Given these changes, only a few studies have analyzed how they may occur. As pointed out by some participants in a study by Moore et al., the mere physical presence of PSWs already motivates certain attitude changes in MHWs [43]. Furthermore, it is generally reported that PSWs intervene in professional groups more directly by introducing the voice of the users, thereby often confronting the professionals’ procedures or attitudes and representing or personifying these changes [51]. This kind of critical work of PSWs is sometimes referred to as a “corrective function” [52]. In professional contexts that lack self-criticism, this kind of work can lead to negative responses and resistance against PSWs, which again hinders the implementation of this workforce [43]. According to a recent study, PSWs could be a role model not only for users but also for MHWs [32].

Against this background, this study aimed to explore the change in attitude that may occur once peer support is implemented in mental health care teams. In this sense, this paper aims to answer two questions: 1) How does the implementation of PSWs in mental health care teams affect the attitudes of MHWs and the larger institutional culture, and 2) how is this change mediated or generated? These questions will be addressed from the perspective of MHWs, as they are at the center of these changes, followed by a discussion on how these changes may contribute to the successful implementation of PSWs.

## Methods

### Study context

The study is reported according to the COREQ checklist Consolidated Criteria for Reporting Qualitative Research). Our research is part of a larger project (ImpPeer-Psy5), exploring the implementation of peer support work in the German mental healthcare system. There are considerable variations in PSW implementation across regions and institutions in Germany, reflecting the diversity of local, regional, and federal regulations and conditions. Public mental health care in Germany is mainly provided through psychiatric hospitals, with approximately 600 hospitals across the country. These hospitals primarily offer inpatient services, followed by a smaller proportion of day clinics, outpatient services, and a very small portion of home treatment services. The services provided by psychiatrists and psychologists/psychotherapists are also regulated by the Fifth German Social Code Book (SGB V) and funded by approximately 90 statutory health insurance companies. Approximately 70% of all expenditure spent on individuals with mental health diagnoses accounts for this sector, which is referred to as the ‘mental healthcare sector’ to differentiate it from the ‘psychosocial sector.’ The latter includes a wide range of complementary, vocational, residential, and psychosocial services regulated by different Social Code Books (mainly SGB IX and XII) and funded by various bodies, such as pension funds or individual social benefits.

The development of the “Experienced Involvement” (EX-IN) training courses for PSWs in Germany was supported by EU funding in 2005. These courses train individuals with lived experiences of mental health diagnosis to become PSWs. Currently, there are 29 EX-IN training sites across the country, and graduates of these programs are certified as PSWs, with their qualifications being recognized by a wide range of mental health service providers. While there are alternatives to EX-IN training, such as peer counseling courses offered by the German Network for Independent Living, EX-IN remains the dominant training paradigm. This led to our study, which aimed to recruit PSWs with varied training backgrounds, being largely dominated by EX-IN graduates due to their greater numbers. While there are no precise data, it is estimated that the majority of the approximately 2500 PSWs in Germany are employed in the latter (SGB IX and XII). However, this study specifically evaluated the implementation of PSW in the mental healthcare sector, primarily focusing on PSWs employed in psychiatric hospital departments, due to the aim of the study and its funding body.

### Study design

The methodological design and processes have been recently published [53], justifying the short description in this manuscript. The ImpPeer-Psy5 study used a mixed-method QUAL1-QUAN-QUAL2 design to incorporate diverse methodological approaches and incorporate various perspectives into exchange. Two qualitative parts of the study (QUAL1 and QUAL2) were conducted at the Brandenburg Medical School (MHB), on which this study is based. An ethics vote was obtained for the Impeer-Psy5 study at Brandenburg Medical School (No. E-01-20200826).

For the QUAL1 phase, a collaborative research approach was used, meaning the cooperation of researchers with and without lived experiences with psychiatric care, mental crises, and recovery from them in all research phases and various educational backgrounds. To further explore and deepen the findings from the QUAL1 (and QUAN) phase, various sub-studies were conducted during the QUAL2 phase, one of which focused on the change in MHWs attitudes following the implementation of peer support work. Thus, this sub-study was mainly accomplished by a psychiatrist (GRP), yet also by drawing on collaborative expertise, as its findings were discussed in the overall collaborative research team.

### Sampling and recruitment

Participants in both the QUAl1 and QUAL2 phases were recruited with the assistance of the primary peer support work training network in Germany, EX(perienced)-IN(volved), and from smaller peer support organizations and projects such as EUTB, Lebensart Münster, and UPSIDES. Information on the study was also disseminated on social media platforms dedicated to peer support-related topics to broaden the pool of recruitment. A total of 57 participants, comprising 32 PSWs, 19 MHWs, and six service users, were recruited for the QUAL1 phase. Given the research focus of the QUAL2 phase on MHWs’ attitudinal changes, eight representatives from this group were recruited to implement both interviews and a focus group. Four of them were also participants of QUAL1. The remaining QUAL2 participants were selected using a combination of snowball sampling and purposive case selection. Participants were not informed of the study prior to the invitation. Information about the research team was provided in the invitation and at the beginning of each interview and the focus group.

### Data collection and analysis

Details of the data collection and analysis can be found in the methodological publications mentioned above [53]. The QUAL1 phase was conducted from 1^st^ February to July 30, 2021, and the QUAL2 phase from 1^st^ September to December 15, 2022. To facilitate a dialogical interview setting, the interviews of QUAL1 were conducted in pairs, comprising two team members: one with and one without lived experience. Interviews of QUAL2 were conducted by one interviewer (GRP), except for the focus group, which was accomplished in pairs (GRP and MK). Interviews took place online due to the COVID19 situation and had an average duration of one hour. The interviews were recorded, transcribed, and analyzed iteratively by a single team member and by the entire research group. Based on the central themes and questions that emerged during this QUAL1 phase, an interview guide was developed for the QUAL2 phase, including the main and sub-questions, such as: What does the implementation of PSWs in psychiatry means for you? What do PSWs contribute to the understanding of psychiatry? What is the PSWs’s understanding of psychiatry and mental illnesses? Do PSWs contribute to paradigm changes in mental health? (S2 File—Interview guide).

These and other questions served to implement five interviews and one focus group by GRP, supported in the case of the focus group by one researcher with lived experience (MK) who also helped with the recruitment and data analysis. No other participant was involved in the assessments. Both the interviews and focus group took place online and were recorded, transcribed, and analyzed both inductively creating new codes and deductively using some codes from the QUAL1 phase, thereby using a modified version of thematic analysis adapted for collaborative research [54]: first, the QUAL2 material was coded inductively. The emerging codes were merged with suitable codes from the QUAL1 phase code tree (S1 File–Code system), and the transcripts of both the QUAL1 and 2 phase were recoded. Data were managed and analyzed using MAXQDA software. Based on the merged code tree and the discursive process carried out between the members of the collaborative research team, thematic analysis was completed, organizing the main codes and subcodes in thematic groups. Transcripts and codes were not returned to the interviewees. In this paper, only the codes that refer to the changes in attitudes of MHWs are presented, and other parts of the analysis will be presented in subsequent papers. We understand attitude as the disposition to evaluate and/or, afterwards, act according to this evaluation in a certain way in response to a specific event, which is influenced by past experience, is not necessarily conscious, and generally serves as the way each person deals with reality and its immediate future. Therefore, attitudes may not be interpreted as perceivable behavior resulting from particular situations but primarily as an axiology, that is, as a personal set of values intervening in and evaluating the situation from this determined standpoint. The theoretical background of our concept will be presented in the framework text (in review).

## Results

All participants provided written informed consent to participate in the study. Sociodemographic information of the participants was gathered on an online survey platform (SosciSurvey) and is illustrated in Tables 1 and 2. Based on the research question and the above-described steps of thematic analysis, the material was divided into two main chapters. The first chapter refers to the attitudinal changes mentioned by the study participants as having resulted from their interactions with PSWs, whereas the second chapter focuses on the experiences that led to these changes.

**Table 1:** Demographic information QUAL1.

QUAL 1		
	PSWs	MHWs
**Gender**		
w	26	5
m	5	10
other	0	0
Total	32	19
**Age**		
< 20:	0	0
20–30:	0	2
31–40:	9	4
41–50:	8	4
51–60:	13	7
61–70:	2	2
>71:	0	0
Total	32	19
**Work as PSWs (years**		
>1	2	
1–3	17	
3–5	7	
5–7	2	
7–9	2	
> 9	2	
Total	32	
**How many years working with PSWs**		
1–5		5
5–10		10
> 10		4
Total		19
**Qualifications**		
EXIN	27	
Other	5	
Total	32	
**Region (Germany)**		
Baden-Württemberg	3	1
Bayern	1	1
Berlin	4	1
Brandenburg	2	2
Bremen	3	2
Hamburg	1	2
Hessen	3	0
Mecklenburg-Vorpommern	1	0
Niedersachsen	5	1
Nordrhein-Westfalen	4	4
Rheinland-Pfalz	1	3
Saarland	0	0
Sachsen	0	1
Sachsen-Anhalt	0	0
Schleswig-Holstein	1	0
Thüringen	1	1
Germany (nationwide)	1	0
Abroad	1	0
Total	32	19
**Profession**		
Psychiatrist		0
Social worker		3
Nurse		10
Psychologist		3
Other		3
Total		19
**Healthcare sector**		
Inpatient	12	10
Outpatient	7	6
Ward-equivalent psychiatric treatment	4	1
Other	9	2
Total	32	19

**Table 2:** Demographic information QUAL2.

QUAL 2	
	MHWs
**Gender**	
w	6
m	2
other	0
Total	8
**Age**	
< 20:	0
20–30:	0
31–40:	3
41–50:	1
51–60:	4
61–70:	0
>71:	0
Total	8
**How many years working with PSWs**	
1–5	2
5–10	3
> 10	3
Total	8
**Profession**	
Psychiatrist	4
Social worker	1
Nurse	1
Psychologist	0
Other	1
Total	8
**Region (Germany)**	
Hamburg	1
Bremen	2
Brandenburg	2
Nordrhein-Westfahlen	2
Total	8
Healthcare sector	
Providers (in- and outpatient)	7
Policy making	1
Total	8

### The “What”: PSWs’ impact on MHWs’ attitudes

#### Taking the user’s experiences more seriously.

Participants generally agreed that implementing peer support expanded the knowledge and perspectives of MHWs by incorporating their unique experiences and insights.

“For me personally and also for my team [it is important] that we take a different perspective, that we also deal with the disorders in a more respectful way, because now there is not only a disorder, but there is also a person with this disorder.” (MHW, interview, QUAL1)

The primary advantage of incorporating PSWs is the shift in perspective. This shift recognizes that caregiving is not solely the domain of professionals, but also encompasses the subjective experiences of individuals recovering from mental crises.

“What has changed? I actually think, paradoxically, it has become a bit clearer to me how it feels from the inside of people to get a diagnosis.” (MHW, interview, QUAL 2)

Working with PSWs allows MHWs to prioritize the needs and perspectives of users, which enables them to access and comprehend the experiences of mental crises in a subjective way.

#### More attention to life contexts.

PSWs can bring attention to certain issues that may not have been considered otherwise. Many individuals have acknowledged that collaborating with PSWs encourages MHWs to place greater emphasis on life circumstances and biographical events rather than concentrating solely on symptoms.

“Nevertheless, I believe, as I just said, that peer workers can also help the treatment team as a whole to look less at diagnoses and more at biographies and contexts in which something happens and in which a person behaves.” (MHW, interview, QUAL 2)

Collaborating with PSWs appears to bring users’ life situations and personal histories to the forefront of MHWs’ focus and attention.

#### More appreciative communication.

Some participants expressed dissatisfaction with the technical terminology employed by MHWs and suggested that the introduction of PSWs led to a change in communication with and about users. One MHW indicated that he observed professional teams communicating with each other and with users in a less technical manner in the presence of PSWs. Another MHW corroborated this view and differentiated between what was considered technical and what was considered human.

“I have become increasingly attentive to language, particularly with regard to technical words. I now find myself questioning whether or not I truly require these terms, as psychiatry has a tendency to make human matters overly technical.“ (MHW, interview, QUAL 1)

Hence, teams are encouraged to adopt a more respectful and comprehensive mode of communication with users, which in turn promotes improved interaction, reduces misunderstanding, and fosters greater mutual understanding.

“For instance, this morning there was no peer support worker there either, and language was unfriendly and pejorative, and it was clear that it was about us being the one or there being the other.” (MHW, focus group, QUAL 2)

According to the participants, the technical language used in psychiatry exacerbated the disparity between professionals and users, resulting in a more pronounced separation between the two groups.

#### More flexibility regarding diagnostic categories.

The majority of participants expressed the belief that PSWs facilitated the process of loosening the diagnostic categories. Additionally, MHWs suggested that collaboration with PSWs could lead to a more flexible perspective on diagnoses, viewing them as processes subject to change.

“[PSW’s] It’s just a slightly different attitude than I’ve experienced in other wards, where the diagnosis was never discussed again... where a diagnosis was already something very fixed.” (MHW, focus group, QUAL 2)

PSWs embody the idea that recovery is achievable, suggesting that MHWs and users can envision a future beyond their current struggles. This belief in a positive outcome during challenging times instilled hope and encouragement.

“That’s basically... Yes. That is part of the job, yes, that they report their experience and give the patients this cue of hope and also transfer their own experience to the team in any case. They definitely do it.” (MHW, interview, QUAL 1)

The incorporation of PSWs allows MHWs to shift their attention away from fixed diagnoses, deficits, or symptoms, and instead concentrate on their strengths and resources that can help them navigate their challenges.

#### Increased openness towards MHWs’ mental health issues.

The influence of PSWs on MHWs was substantial in their approach to managing their own crisis experiences. It was discovered that MHWs’ perspectives on professionals’ personal histories of mental health issues changed as a result of their interaction with PSWs. Certain participants expressed a heightened sense of empathy and understanding towards their colleagues.

“We recently had an application from a nurse, and she said at the very end of the interview: ‘But I have to say one more thing, I was a patient with you in psychosomatic medicine six months ago.’ And my boss looked straight into the round and said, That’s not a problem, we will hire you. And I think that a few years ago, without the peer support worker... we would not be here yet.” (MHW, focus group, QUAL 2)

Furthermore, MHWs with mental health struggles may feel more empowered to openly discuss and share their experiences when collaborating with PSWs. Consequently, the involvement can foster increased openness and mutual respect for these experiences.

“This has actually happened to me twice now, that these colleagues approached me and said, now with the peer it is a lot easier for me to talk about my own depression again.” (MHW, focus group, QUAL 2)

Certainly, PSWs play a vital role in blurring the conventional distinction between individuals with and without illnesses, as outlined in Section 3.1.3. This concept is particularly relevant in the context of mental health care, as emphasized by our study’s participants.

### The “How”: how are these changes brought about?

#### PSWs can reinforce staff attitudes towards change.

Several individuals indicated that a significant number of professionals were actively seeking to effect change prior to the introduction of peer support, largely stemming from their dissatisfaction with the mental healthcare system. These participants criticized the system as a “repair shop” that aimed to “fix” disorders that were primarily attributed to medical conditions. Consequently, they embraced peer support work as a further step in transforming it.

“This is the kind of idea that is embedded in our health system: it is a disease, a deficit. And that we must have users become like they were before this disease. Like a repair shop. There is something wrong and we have to repair it.” (MHW, interview, QUAL 1)

Critics have expressed dissatisfaction with the medical affiliation of the mental health care system because of the prevalence of biological perspectives. PSWs have been recognized as allies in the effort to dismantle or alter this undesirable medicalized mental health care system.

“The special thing, I say from my philosophy, is that peer support can contribute pretty good to deconstructing the psychiatric system.” (MHW, focus group, QUAL 2)

#### PSWs as role models.

The use of PSWs can serve as a basis for MHWs to emulate. By working together, the conventional practices can evolve. MHWs may attempt to adopt certain interaction methods that are employed by PSWs. During the QUAL2 phase, one prominent finding involves adopting a more unrestricted approach to engaging with users, which is modeled after the PSWs’ methods and is not restricted by administrative or financial limitations.

“It is sometimes difficult for me to move away from the hospital setting […]. I am forced to document and think of funding in such a way that I repair someone as quickly as possible. I think cooperation with peers has changed my attitude slightly, because it is just not so timed, because they are much more open in their goal setting compared to the rest of our team” (MHW, focus group, QUAL 2).

Thus, PSWs are examples of different ways of interacting, both with other colleagues and with users:

“The peers I know were always very, very professional, they also kept their eyes open for the fact that crises and conditions that are overwhelming are simply part of being human and thus also made it a little easier for the other colleagues to be more open with their human concerns and needs.“(MHW, interview, QUAL 1)

#### Presence of PSWs.

It was noted that the mere presence of PSWs influenced the participants as they symbolized or represented users’ perspectives or needs.

“I think it’s more about hearing things through the patient’s ear. It is easier to think about what patients would think if they heard it themselves, or something like that, if there were something like placeholders or representatives. I think this happens automatically and unconsciously.” (MHW, interview, QUAL 2)

The shift in language used by MHWs is a clear and recurring example, as previously discussed. This change is likely influenced by the PSWs acting as the patient’s ear, resulting in MHWs referring to them with increased respect.

“The language is more restrained and that could have something to do with the fact that you have the feeling that people are listening and talking to you who might feel affected in a bad way if you talk like that” (MHW, interview, QUAL 2)

PSWs may serve a preventive function not only with regard to the language used, but also in the context of coercive measures.

“… it is important especially when there are coercive measures in the room or when patients are in the isolation room or are being restrained, that someone who knows this automatism in psychiatric wards from the inside, is there as a corrective perception or to plea for understanding for the position of the person concerned.” (MHW, interview, QUAL 1)

In the context of mental healthcare practices, the unconscious automaticity of some practices regarding violence and coercion is often reflected in the presence of PSWs. Furthermore, our participants identified similar dynamics in the power relationships between MHWs and users.

#### Direct feedback and critique.

Ultimately, it is possible for shifts in the attitudes to arise from feedback provided by PSWs. Such feedback can make MHWs aware of certain “automatisms” of which they were not previously conscious.

“An example: there was a ward round and the senior physician said something ironic or sarcastic to a patient, and the patient didn’t understand it at all and became very aggressive. Afterwards the peer explained in the debriefing that during a psychosis one is absolutely incapable of understanding sarcasm.” (MHW, focus group, QUAL 2)

Through this kind of direct intervention, PSWs can “open the eyes” of MHWs hitherto not be sufficiently considered from users’ perspectives:

“When there is a senior doctor’s round, there are sometimes five or six of us sitting in a room, and the patients come in. We did not understand how overwhelming this tribunal was. And that’s when our peer simply opened our eyes, who said: “it’s really hard when you come in like that and the perceived authorities are sitting there.” As a result, we changed our setting. We have formed a circle so that the patient sits with us.“ (MHW, interview, QUAL 1)

However, certain professionals have suggested that providing direct feedback to MHWs may elicit negative reactions that could hinder the integration of PSWs into the team.

“It takes a lot of courage to report that back, to say to someone, listen, so the way you are talking about this person right now, that does not sound very appreciative to me. This takes a lot of courage and, in turn, also needs the support of the management, that is, the team leader, not only my support but also the team leadership. If that doesn’t fit and the peer support worker has no support, no reinforcement, then unfortunately that’s difficult.“(MHW, interview, QUAL 1)

It is clear to our participants that it takes a great deal of courage for PSWs to perform this function, and that the support of the institution’s management level is indispensable.

## Discussion

According to our findings, collaborating with PSWs may have a significant impact on the attitudes, leading them to incorporate insights from PSWs’ experiences, consider life circumstances, adopt a more empathetic communication style, employ diagnostic categories with greater flexibility, and exhibit greater receptiveness towards their own and other MHWs’ mental health issues. Moreover, these transformations may be facilitated either by the mere presence of PSWs, acting as role models for other staff, or by more direct forms of feedback, intervention, and critique.

### What is changing?

The existing body of research demonstrates that PSWs have a favorable influence on treatment outcomes [10]. However, as previously noted in the introduction, the specific impact of PSWs on the attitudes of MHWs and the broader institutional culture has not been specifically explored.

Most of our results suggest that the presence of PSWs may encourage a greater recovery orientation among MHWs, a finding consistent with other studies [8,55] and reviews [30,40]. Thus, the MHWs in our study report that working with PSWs makes them pay more attention to the contextual aspects of the users’ lives, which are often overlooked. Users’ experiences of mental crises have become more important to them. In this context, Gillard used the term “body of practice” to frame this kind of impact of PSWs on mental health care teams, thereby increasing their skills [25]. Thus, being better able to understand the user’s experience, MHWs may acquire a perspective of care that goes beyond a mainly diagnostic gaze, instead focusing more on a person’s life context. Moore et al. refer to something similar when they talk about “the ‘little’ things” that PSWs are able to give voice to – aspects that are important to users that are easily overlooked [43].

Another outcome concerns the communication and use of language by MHWs. In our interviews, the presence of PSWs motivated more cautious use of language, leading to a more appreciative and less stigmatizing manner. This not only refers to situations with users but also to situations in which MHWs communicate with each other, in which the vocabulary used usually tends to be less carefully chosen. The language was changed and became less technical, as described by Hollway et al., adding that such a technical language usually serves as an emotional barrier against users, as a kind of defense mechanism on the part of MHWs [56]. In an alternative interpretation, the use of managerial or medical language by MHWs is a symbolic scenario that reflects power dynamics in a discursive manner [57]. In our interviews, MHWs’ ‘technical’ ways of thinking and speaking were perceived as a means of maintaining the distinction between ‘them and us’ or ‘sick and healthy,’ potentially resulting into a lack of engagement with users or their exclusion [43]. In this way, the presence of PSWs seems to improve communication with users, promoting better mutual understanding, a finding that has also been presented by Eassom et al. [45] and other researchers [46].

Another result that leads to greater recovery orientation in professional teams is greater flexibility when using diagnostic categories. However, we did not find this aspect in the literature. With this, some participants tried to compare situations with and without PSWs, where in the latter, many diagnoses tended to be considered static or fixed. On the other hand, PSWs are an example of how, on the one hand, diagnostic categories evolve and that users are not only defined by the symptomatic deficits that define them; on the other hand, they serve as hope-givers for users and professionals, as they represent a history of recovery. Finally, these changes in attitude can lead to institutional changes beyond individuals. A more recovery-oriented institutional culture can be a favorable environment that allows MHWs to gain more insight into their own affectedness. As described by Byrne et al., PSWs can motivate disclosure regarding their own questions in relation to the well-being and psychological crises of professional staff [50]. This again clashes with the usual position of professionals who traditionally stand on one side of the divide vis-à-vis users, who are the ones who are affected by mental problems. As discussed earlier, a greater culture of disclosure has the potential to loosen this divide [49].

Various authors have demonstrated that the implementation of peer support work highly depends on how PSWs and other MHWs relate to each other, understanding the success or failure of peer support implementation as emerging from a complex interplay of power imbalances between these professional groups [47]. In this context, as stated above, medicalized attitudes based on the predominance of fixed diagnostic categories and psychopharmacological approaches seem to be a dominant obstacle to mutual understanding between them and PSWs [21,23]. Thus, the change in attitudes of MHWs is pivotal to the implementation of peer support work, opening up the dilemma of when these changes must occur to make this implementation happen, a dilemma that will be further detailed in the concluding section.

### How change happens?

Our findings show that MHWs’ attitudes typically undergo transformations due to reflective processes that follow their interactions with PSWs. Our research uncovered three ways in which these reflections occur: initially, the mere “physical presence” of PSWs appears to trigger change, as also described by some participants in Moore et al.’s study [43], who reflected on uncomfortable emotional responses on the occasion of PSW’s “simple physical presence”. This process may occur explicitly or implicitly so that changes may also occur without the individual being consciously aware of them. Second, our study participants cited a “role-modeling” function for PSWs, which also led to imitating PSWs’ approach to users. Although the literature usually emphasizes this function only in relation to users of mental healthcare services, it also holds true for MHWs [25,58]. Krumm et al. also found this point reported directly from MHWs [32]: PSWs exemplify a specific attitude or approach, and MHWs adopt this style into their own repertoire. Third, PSWs appear to elicit attitudinal changes in MHWs through direct explicit forms of feedback or critique. The literature indicates that PSWs do not necessarily have to say much, as their hybrid role - as former users employed in mental health care teams - already challenges the usual routines to a considerable extent [51]. In accordance with our findings, other studies describe this function as “corrective,” which may trigger resentment and other strong emotions among MHWs [52]. As a result, PSWs often have to contend with disinterest, stigma, rejection, ignorance, and discrimination from colleagues.

We argue that MHWs’ attitudinal transformations are rooted in reflexive processes. As indicated by our research and other studies [41], the successful implementation of these changes requires openness and willingness to change on the part of MHWs. Consequently, MHWs who are open to collaborating with PSWs are typically more open to altering their prior perspectives on mental health care, particularly their role as a ‘repairer’ of biological disruptions. It appears that this mindset is more common among MHWs who are more open to their own experiences of crisis and/or recovery [20]. Our study also highlights these interactions and the role of self-criticism in facilitating change: some MHWs criticize the current system, perceiving the implementation of PSWs as a further step toward a larger transformation towards a more recovery-oriented mental healthcare system [21]. A risk that should be mentioned here is that in institutions where resistance to attitudinal changes is greater either because of little experience with PSWs or because of a greater weight of the medical model, PSWs may end up integrating into the traditional structure and adapting to it, thus reinforcing the power structures based on this medicalized model [59].

## Limitations

Most of the limitations of our study are inherent to the qualitative research undertaken. A restricted sample size may hinder the study from reflecting the diverse structure and aims of these roles. This study only recruited MHWs for the QUAL2 phase because of the focus on their attitudinal changes. These factors restrict the generalizability of our findings to a broader population of PSWs and MHWs. However, the absence of one-sided trends in the data indicates diversity within both professional groups. Finally, due to purposive sampling, it is likely that a positive bias was generated among the participants regarding the work and implementation of PSW, as well as a generally favorable opinion towards the recovery philosophy in mental health.

## Conclusion. Chicken and egg problem: When must change occur?

In our search for literature to contextualize our findings, we discovered that numerous studies emphasize the need for changes in MHWs and the broader institutional culture as a prerequisite for the successful implementation of PSWs. However, most studies pay little attention to the mechanisms through which these changes may occur. In our study, we found that changes were primarily mediated by direct contact between PSWs and MHWs through their mutual presence through processes of role modeling or direct feedback. This presents a dilemma: what can be done when the changes that are necessary to prepare for the successful implementation of PSWs are primarily brought along through direct contact with this workforce, that is, only after it has already been implemented?

One usual method for preventing challenges or fostering acceptance of PSWs in mental health care settings is to provide training to teams before their implementation [33,58,60]. Collaborative training conducted by trainers with and without lived experiences appears to be crucial in relation to this dilemma, as they allow for direct contact with PSWs prior to their implementation [39]. Additionally, internships for MHWs to observe the work of PSWs in another clinical setting may be important for preparing for their acceptance. Finally, it may be beneficial to see peer support work implementation as a gradual process that unfolds over time. The integration of PSWs within teams typically evolves progressively as mindsets shift slowly, and team members gain a deeper understanding of their roles [3,61]. Changes may not occur rapidly and may require conflicts and dissonance, which, in the case of our study, may often only begin to subside after the first year of PSW implementation.

Thus, answers to the question of whether individual or institutional change should occur before or after the implementation of PSW remains somewhat ambiguous. This change is necessary before and after implementation, forming an ongoing loop that continually strives for the transformation of MHWs’ attitudes and broader institutional culture through sustained collaborative experiences. While PSW implementation requires a willingness to change on the part of MHWs, which can be fostered through training and direct interaction, actual transformation primarily occurs through collective teamwork, where collaboration is continuously developed and refined.

### Consent for publication

All participants provided formal consent and approved participation and publication of the results.

## Supporting information

S1 File Code system. (DOCX)

S2 File Interview guide. (DOCX)

## Abbreviations

MHWs: mental health workers (plural)
MHW: mental health worker (singular)
PSWs: peer support workers
QUAL: qualitative phase
QUAN: quantitative phase
